# Supplementary material for: Getting More Out of Biomedical Documents with GATE's Full Lifecycle Open Source Text Analytics
Source: PLoS Comput Biol. 2013 Feb 7;9(2):e1002854. doi: 10.1371/journal.pcbi.1002854 (PMC3567135; doi:10.1371/journal.pcbi.1002854)
Supplement: Dataset S1 — GATE software. Dataset S1 bundled with this paper contains a distribution of GATE (or see http://gate.ac.uk/download/). (TGZ) [file pcbi.1002854.s001.tgz › plos-gate/plugins/Ontology/test/ontologies/w3tests/copyright-software-20021231.html]

W3C Software License

# W3C^�^ SOFTWARE NOTICE AND LICENSE

### http://www.w3.org/Consortium/Legal/2002/copyright-software-20021231

This work (and included software, documentation such as READMEs, or other
related items) is being provided by the copyright holders under the following
license. By obtaining, using and/or copying this work, you (the licensee)
agree that you have read, understood, and will comply with the following
terms and conditions.

Permission to copy, modify, and distribute this software and its
documentation, with or without modification, for any purpose and without
fee or royalty is hereby granted, provided that you include the following on
ALL copies of the software and documentation or portions thereof, including
modifications:

1. The full text of this NOTICE in a location viewable to users of the
   redistributed or derivative work.
2. Any pre-existing intellectual property disclaimers, notices, or terms
   and conditions. If none exist, the W3C Software Short
   Notice should be included (hypertext is preferred, text is permitted)
   within the body of any redistributed or derivative code.
3. Notice of any changes or modifications to the files, including the date
   changes were made. (We recommend you provide URIs to the location from
   which the code is derived.)

THIS SOFTWARE AND DOCUMENTATION IS PROVIDED "AS IS," AND COPYRIGHT HOLDERS
MAKE NO REPRESENTATIONS OR WARRANTIES, EXPRESS OR IMPLIED, INCLUDING BUT NOT
LIMITED TO, WARRANTIES OF MERCHANTABILITY OR FITNESS FOR ANY PARTICULAR
PURPOSE OR THAT THE USE OF THE SOFTWARE OR DOCUMENTATION WILL NOT INFRINGE
ANY THIRD PARTY PATENTS, COPYRIGHTS, TRADEMARKS OR OTHER RIGHTS.

COPYRIGHT HOLDERS WILL NOT BE LIABLE FOR ANY DIRECT, INDIRECT, SPECIAL OR
CONSEQUENTIAL DAMAGES ARISING OUT OF ANY USE OF THE SOFTWARE OR
DOCUMENTATION.

The name and trademarks of copyright holders may NOT be used in
advertising or publicity pertaining to the software without specific, written
prior permission. Title to copyright in this software and any associated
documentation will at all times remain with copyright holders.

\_\_\_\_\_\_\_\_\_\_\_\_\_\_\_\_\_\_\_\_\_\_\_\_\_\_\_\_\_\_\_\_\_\_\_\_

This formulation of W3C's notice and license became active on December 31
2002. This version removes the copyright ownership notice such that this
license can be used with materials other than those owned by the W3C,
reflects that ERCIM is now a host of the W3C, includes references to this
specific dated version of the license, and removes the ambiguous grant of
"use". Otherwise, this version is the same as the previous
version and is written so as to preserve the Free
Software Foundation's assessment of GPL compatibility and OSI's certification
under the Open Source
Definition. Please see our Copyright FAQ for
common questions about using materials from our site, including specific
terms and conditions for packages like libwww, Amaya, and Jigsaw. Other
questions about this notice can be directed to site-policy@w3.org.

Joseph Reagle <site-policy@w3.org>

Last revised $Id: copyright-software-20021231.html,v 1.11 2004/07/06 16:02:49 slesch Exp $
